# Detecting truly clonal alterations from multi-region profiling of tumours

**DOI:** 10.1038/srep44991

**Published:** 2017-03-27

**Authors:** Benjamin Werner, Arne Traulsen, Andrea Sottoriva, David Dingli

**Affiliations:** 1 Centre for Evolution and Cancer, The Institute of Cancer Research, London SM2 5NG, UK; 2 Department of Evolutionary Theory, Max Planck Institute for Evolutionary Biology, 24306 Plön, Germany; 3 Division of Hematology and Department of Internal Medicine, Mayo Clinic, Rochester, MN, USA; 4 Department of Molecular Medicine, Mayo Clinic, Rochester, MN, USA

## Abstract

Modern cancer therapies aim at targeting tumour-specific alterations, such as mutations or neo-antigens, and maximal treatment efficacy requires that targeted alterations are present in all tumour cells. Currently, treatment decisions are based on one or a few samples per tumour, creating uncertainty on whether alterations found in those samples are actually present in all tumour cells. The probability of classifying clonal versus sub-clonal alterations from multi-region profiling of tumours depends on the earliest phylogenetic branching event during tumour growth. By analysing 181 samples from 10 renal carcinoma and 11 colorectal cancers we demonstrate that the information gain from additional sampling falls onto a simple universal curve. We found that in colorectal cancers, 30% of alterations identified as clonal with one biopsy proved sub-clonal when 8 samples were considered. The probability to overestimate clonal alterations fell below 1% in 7/11 patients with 8 samples per tumour. In renal cell carcinoma, 8 samples reduced the list of clonal alterations by 40% with respect to a single biopsy. The probability to overestimate clonal alterations remained as high as 92% in 7/10 renal cancer patients. Furthermore, treatment was associated with more unbalanced tumour phylogenetic trees, suggesting the need of denser sampling of tumours at relapse.

Recent advances in next-generation sequencing have led to the widespread identification of somatic changes in the genomes of a large number of tumours, raising the hope to transform cancer therapy based on patient-specific data^1^. Novel treatments aim at targeting cancer genomic alterations, or prime the immune system to neo-antigens expressed by tumour cells, allowing personalised cancer medicine^2^,^3^,^4^,^5^,^6^,^7^,^8^,^9^,^10^,^11^.

The success of this therapeutic strategy however, relies on selecting the correct targets in each patient^8^,^12^,^13^,^14^. The number of potentially targetable tumour specific alterations is continuously increasing. However, any approach that targets sub-clonal alterations will at best eradicate only a proportion of cells in the tumour. For a maximal effective therapy (and any prospect of tumour eradication), tumour-specific alterations that are present in all cells of the tumour and thus are “truly” clonal must be targeted by therapy^13^,^15^,^16^,^17^.

However, intra-tumour heterogeneity and sampling bias complicate the correct classification of truly clonal and sub-clonal alterations. Independent multi-region profiling of spatially distinct tumour samples increases the information on individual tumours and allows the reconstruction of phylogenetic trees^18^,^19^,^20^,^21^,^22^,^23^,^24^,^25^,^26^. Truly clonal alterations must appear in the “trunk” of these trees. However, the opposite is not necessarily true. An alteration that appears truncal in the “sampled” tree, may still be sub-clonal in the whole tumour because we cannot profile every cell in the neoplasm^23^,^27^, see also Fig. 1. Taking larger, more or spatially distant samples can mitigate the problem^19^,^22^,^23^,^24^,^25^, but the fundamental question remains: how many samples of a tumour do we need to identify the list of all truly clonal alterations with a certain confidence?

## Results

**Figure 1:**
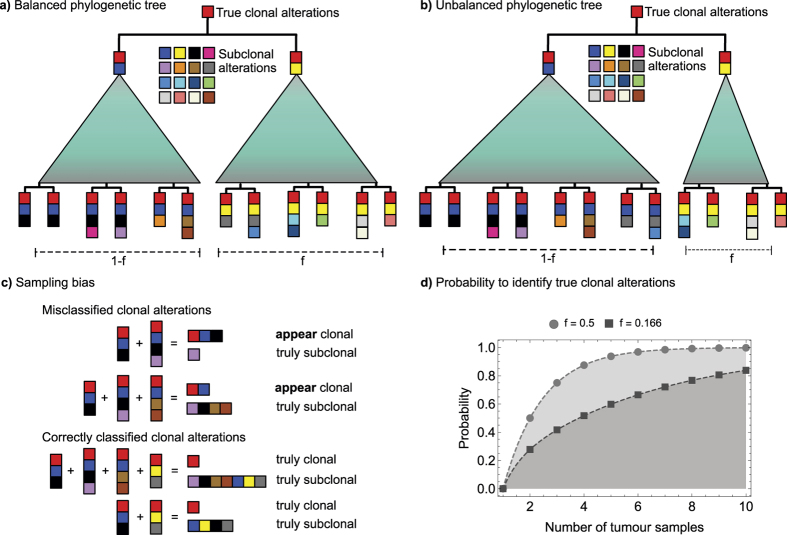
The sampling bias of a multi region analysis depends on a tumour’s evolutionary history. (**a,b**) The most recent common ancestor of all cells in the tumour contains all alterations that are truly clonal (top square). The first bifurcation from the ancestor divides the tumour into two populations that will constitute a fraction of *f* and 1 − *f* at diagnosis. These fractions are the result of complex processes (e.g. clonal selection) and tumours might be balanced (both populations reach a similar size, *f *= 0.5), or one population gains a significant fitness advantage and the tumour becomes unbalanced (*f* ≪ 0.5). During growth, cells accumulate further alterations that contribute to intra tumour heterogeneity at diagnosis. (**c**) This implies that different multi-region samples will identify different alterations and different combinations of samples will identify different sets of clonal and sub-clonal alterations. Only if we sample cells from both sides of the phylogenetic tree, we can identify all true clonal alterations. (**d**) The probability that at least one out of *i* samples is from each side of the phylogenetic tree depends on the relative sizes of both sides *f* and is given by *p*_*f*_ = 1 − *f*^*i*^ −(1 − *f*)^*i*^. Balanced trees (*f *= 0.5) need few samples to identify all true clonal mutations with high confidence, while unbalanced trees (e.g. *f *= 0.166) require more samples for the same confidence.

Let us consider the complete phylogenetic tree of a tumour. Each leaf of this tree is a cancer cell. Leaves are separated by bifurcations representing cell divisions prone to inheritable alterations, which could be single nucleotide polymorphisms, gene duplications, translocations or any other genomic change. Alterations that are in the trunk of the tree must be present in all cells of the tumour, if we neglect unlikely events of back mutations. The first bifurcation divides the tumour into two populations of fraction *f* and 1 − *f*. The sizes of these fractions are the result of potentially complicated processes, e.g. clonal selection, immune system escape or random drift. If we were to sample from both sides of the tree, all alterations that appear clonal in both samples will also be truly clonal in the whole tumour. But if we only sample from either side, we will misclassify a fraction of sub-clonal alterations as clonal, see Fig. 1. Thus the critical question is, how likely are we to sample from both sides of the tree in a multi-sampling strategy? Assuming we analysed *i* independent spatially separated tumour samples, the probability to sample from both sides of the tree is


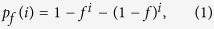


see Methods for details. The information gained from multi-region sequencing follows a single universal curve and the balancing factor *f* determines the shape of this curve, see Fig. 1d. The probability to classify all truly clonal alterations correctly from a single sample is expected to be zero (*p*_*f*_(*i *= 1) = 0). Including more samples *i* to the analysis increases the probability to classify truly clonal alterations correctly. The probability increases fastest for trees in which the first bifurcation splits the tumour population approximately in half (*f *= 1/2). These are often referred to as ‘balanced’ phylogenetic trees, and are often, but not always, consistent with neutral growth (i.e. all the tumour driving alterations were present in the trunk of the tree)^27^. In this case, the information is gained exponentially 
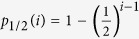
 with the number of samples *i*. Two tumour samples have a probability of 50% to correctly classify all truly clonal alterations and the probability increases to 99% for 8 independent samples. However, the probability increases more slowly in unbalanced tumours, e.g. in cases of strong on-going sub-clonal selection during tumour growth or as a result of treatment. For example, if one side of the tree is 5 times larger compared to the other side, two independent tumour samples result in a probability of 28% to correctly classify all alterations and increases to 73% for 8 independent samples (Fig. 1). Given that the spatial distribution of mutations in the tumour cannot be known a priori, there cannot be a unique sampling strategy, as different tumours might present with different relative *f* and the uncertainty to identify truly clonal alterations might be dramatically different for two patients with the same number of samples. Ideally, the sampling strategy should be adjusted to account for each tumour’s individual evolutionary trajectory.

The balancing factor *f* can be inferred from multi-region profiling of individual tumours, see Methods for details. In short, comparing the lists of clonal alterations identified by all permutations of tumour samples gives a measure for the average information gained by additional sampling. This information gain should fall onto the universal curve (1) after adjusting for finite sampling (see Equation (8) in the Method section for details). For example, if we have 10 tumour samples in total, we can generate 45 unique combinations of 2 subsamples. If the tumour were perfectly balanced (*f *= 0.5), half of the subsample combinations would recover the exact minimal list of clonal alterations. For unbalanced tumours (*f *< 0.5) fewer combinations of subsamples will recover the minimal list of alterations. This procedure is then continued for all possible combinations of subsamples. Comparing the shape of the universal curve (8) to the actual information gain from the data allows assigning an empirical balancing factor *f* to a tumour. Each tumour-specific balancing factor provides a rational of whether the current number of tumour samples is sufficient, or if additional sampling is necessary to ascertain the identity of truly clonal alterations in that particular patient. In addition, the value of *f* would determine whether it makes sense to sequence additional parts of the tumour, if the expected information gain from each sample is very small.

First, we tested if the information on clonal alterations gained from multi-region sequencing data falls onto the theoretically predicted universal curve (8). We evaluated ten cases of multi-region sequenced clear cell renal carcinoma (between 5 and 11 samples per tumour, 74 samples in total) recently published by Gerlinger *et al*.^18^,^22^. Each sample had a volume of approximately 0.25*mm*^3^ and thus each sample contained ~10^8^ cells. The protein coding region of the genome (exome) was sequenced with a depth of >70x for all samples, allowing the identification of clonal mutations within each single bulk sample with high precision.

**Figure 2:**
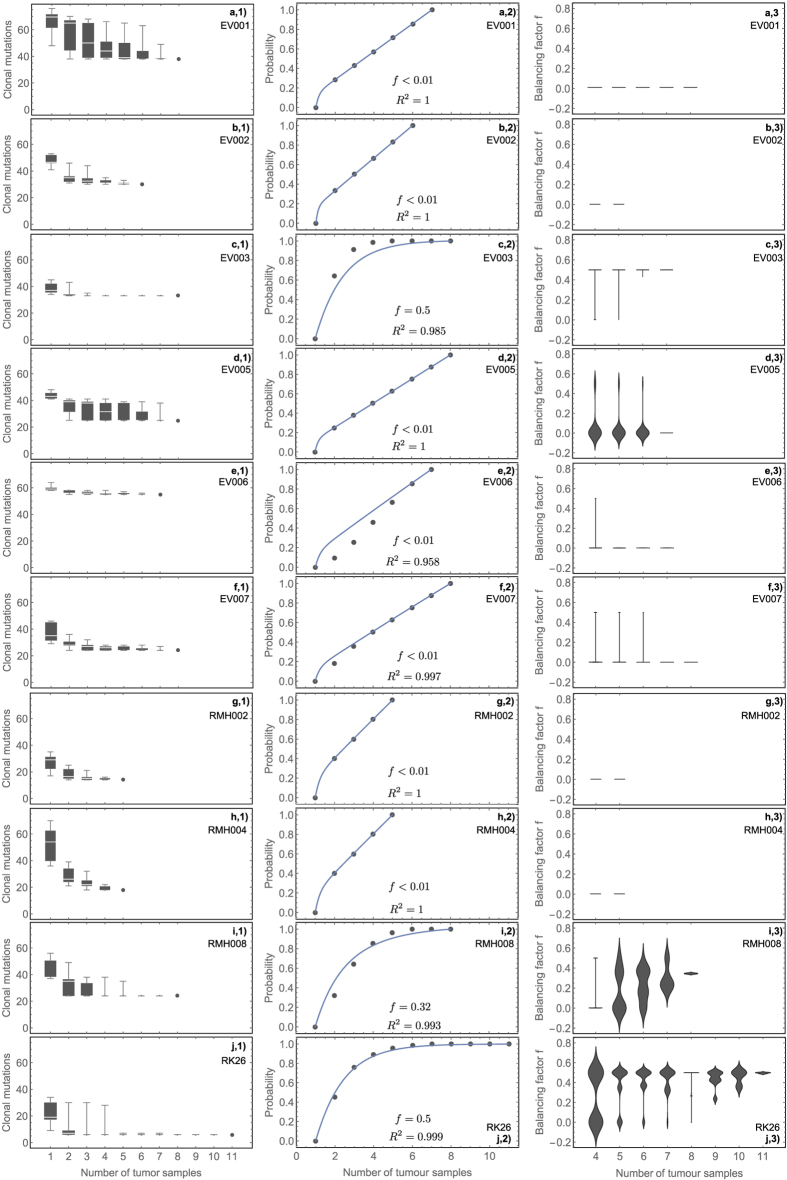
Information gain from multi-region sequencing in patients with clear cell renal carcinoma. (Panels a1 to j1): If from a set of *n* multi-region samples from a patient we consider different subsets of samples (*n* is between 5 and 11 per patient) with size *i *= 1,2,…*n*, we will identify different numbers of putatively clonal alterations, with great variation between different sets of the same size. The more samples we consider, the closer we get to the minimal identifiable set of clonal mutations, i.e. mutations that may have appeared clonal with one or few samples, turn out to be indeed sub-clonal in the whole tumour. (Panels a2 to j2): The probability to find the minimal set of clonal mutations falls onto the universal curve (8). Dots represent the data; lines correspond to best fits of *f* via Equation (8). In 2 cases (c2 and j2) we find a balanced left and right side (*f *= 0.5). One case (**i**) appears slightly unbalanced (f = 0.32) while all other cases are unbalanced (f < 0.01), supporting the presence of convergent evolution and on-going clonal selection. All patients but (i2) and (j2) developed metastasis. Only patients (h2 to j2) are treatment naïve. For balanced tumours, the information on the true set of clonal alterations quickly plateaus with few samples (for example 5 samples in patient (**j**)). (Panels a3 to j3): We repeat the inference of the balancing factor *f* on all available combinations of subsets of tumour samples with a minimum of 4 samples. The violin plots show the corresponding distributions of *f* values for each possible combination of *i *= 4,5,…*n *− 1 subsets. Most combinations of samples resemble the balancing inferred from the full data set. However, there is a trend towards a bimodal distribution for small *i*, which might be a direct consequence of the spatial evolution of tumours. Note that violin plots show the probability density distribution of the *f*-values. The actual *f*-values are never negative. Data from Gerlinger et al.^22^.

Intra-tumour heterogeneity was high in all 10 tumours. The number of coding mutations identified within a single sample ranged from 9 to 76 across tumours, see Fig. 2 panel a1 to j1. Considering more samples in the analysis decreases the number of what appeared to be clonal mutations, as well as the variability in all 10 cases, e.g. 8 samples from the same tumour reduced the list of clonal mutations on average by 40% compared to a single sample and the reduction ranged from 14 to 72% in individual patients, see also Fig. 2 panel a1 to j1.

Strikingly, the universal curve (8) describes the information gain from additional samples very well in all 10 cases and we can assign balancing factors to all 10 tumours. We found balanced phylogenetic trees (*f *= 0.5) in only two tumours, see Fig. 2 panel a2 to j2. In these cases, eight tumour samples suffice to identify all truly clonal mutations with a probability of 99%. One tumour had a slightly unbalanced tree (*f *= 0.35), while 7 tumours appeared to be highly unbalanced (*f *< 0.01). In the latter cases, distinct clonal expansions were likely driven by selection, supporting the original findings of the authors of on-going clonal selection and convergent evolution in the majority of the patients analysed^18^,^22^. In these cases, a study with fewer or different samples on the same tumour would have identified very different sets of clonal mutations. Based on the data, two samples have a median probability of 68% (a 95% CI of 55 to 77%) to overestimate the number of clonal mutations, highlighting the potential risk of suboptimal treatment strategies due to incomplete information on clonal genomic changes of tumour cells. Adding more tumour samples to the analysis of the 7 unbalanced tumours would likely reduce the list of putative clonal mutations further, allowing for a better-informed course of treatment.

We note that the balancing factor *f* was independent from the total number of uniquely detected mutations (Spearman Rho = −0.38, p = 0.3), or the percentage of uniquely detected mutations defined as clonal across all samples of a single tumour (Spearman Rho = 0.18, p = 0.62). The mutational load of a tumour is the result of many potentially interacting factors, e.g. the age of a patient or the intrinsic (potentially elevated) mutation rate. Furthermore the majority of mutations are likely neutral passengers or provide only a weak selective advantage to the tumour and correlations might be masked by treatment induced selection biases. This suggests that a sampling strategy based on mutational diversity alone may not be optimal. As we show, the change of diversity across independent tumour samples is the variable of interest.

We then tested the robustness of our estimates by applying our analysis to a subset of tumour samples. We inferred the balancing factor *f* for all possible combinations of subsets with a minimum of 4 samples. For example, all combinations of 6 out of 12 tumour samples yield 924 independent estimates for *f*. The distributions of values for *f* are summarised in Fig. 2 panel a3 to j3. Most combinations of samples resemble the balancing inferred from the full data set. We observe a trend towards a bimodal distribution for small sample numbers (e.g. Fig. 2 d3, i3 and j3). This might be a direct consequence of the spatial sampling scheme. Few samples in close spatial proximity are more likely to show balanced (neutral) growth characteristics, whereas samples with maximal spatial distance likely diverged early during tumour development^23^,^27^,^28^. This suggests that conclusions about the evolutionary history of tumours based on only a few samples can be misleading. Sufficiently many spatially distant tumour samples are required for a reliable inference (and interpreted in the context of *f*).

Interestingly, 6/7 unbalanced tumours received treatment before resection (and sequencing) and all 7 cases developed metastatic disease. In contrast, 2/3 balanced tumours were treatment naive at the time of sequencing and the only 2 tumours without metastatic disease (Fig. 2i,j) were balanced. Indeed, tree unbalancing was associated with treatment (p = 0.02, t-test), indicating that treatment likely contributes to high selection pressures that lead to unbalanced phylogenetic structures. This has important biological and clinical implications, suggesting that treated tumours may require more samples to design the optimal therapeutic strategy based on truly clonal alterations. In addition, it appears that multi-region sequencing *before* initiation of any therapy may simplify the identification of truly clonal abnormalities that could be the targets of therapy. Future studies are needed to test this observation further. It will also be important to stratify patients for potentially other confounding factors, such as tumour size, tumour stage, and the spatial distribution of tumour samples.

**Figure 3:**
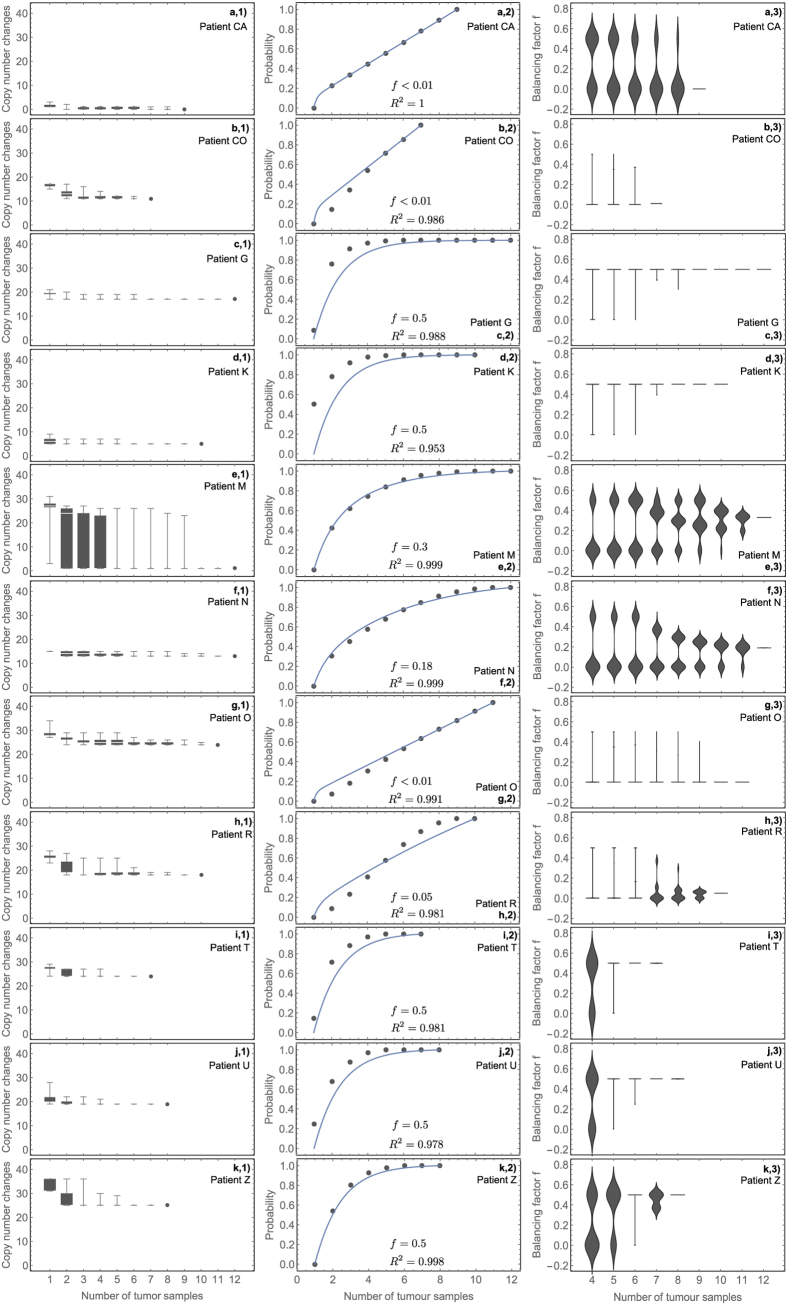
Information gain from multi-region copy number profiling in patients with colorectal cancer. Copy number changes were inferred from spatially distributed single glands of 11 colorectal tumours. Based on the shape of the universal curve (Equation (8)), 7 tumours appear balanced or nearly balanced and 4 tumours appear unbalanced. Balanced tumours require fewer samples to identify truly clonal copy number changes, whereas uncertainty remains high in unbalanced trees. Data from Sottoriva *et al*.^23^.

Next, we tested if the information on copy number changes also follows our theoretical prediction (8). We reevaluated copy number changes in multiple single crypts (each crypt contains ~10^4^ cells) of 11 treatment naive colorectal tumours (7–13 crypts per tumour, 107 samples in total) previously published in ref. 23. Again the information gain from multiple tumour samples is well described by our theoretical model (see Fig. 3 panels a2 to k2). Five tumours are characterised by balanced phylogenetic trees (*f *≈ 0.5), two cases show slightly unbalanced trees (*f *= 0.19 and *f *= 0.3) and four cases have unbalanced trees (*f *< 0.01). Based on this data, two samples have a median probability of 58% (95% CI of 38 to 75%) to overestimate the number of clonal copy number changes. Overall, these results support previous observations of largely a single clonal expansion in a majority of colorectal tumours that would lead to more balanced phylogenetic trees^19^,^27^. In these cases, a few samples can identify truly clonal copy number changes. However, we also identified four cases with an unbalanced phylogenetic history, similar to the 7 cases in renal cell carcinoma. Treatment strategies for these patients might benefit from an analysis of additional samples.

There was no correlation between tumour balancing and the total number of unique copy number changes (Spearman Rho = 0.16, p = 0.63). However, we observed a strong positive correlation between the balancing factor *f* and the percentage of unique copy number changes (Spearman Rho = 0.76, p = 0.007). Balanced tumours (*f *≈ 0.5) acquired fewer sub-clonal copy number changes (relative to the number of clonal copy number changes) compared to unbalanced tumours. This is in contrast to the mutational burden in renal cancer patients, where we could not observe a similar correlation. There are several potential reasons for this observation. All colon cancer samples were treatment naive. Copy number changes occur less frequently compared to mutations and do not accumulated with age in healthy tissues. Furthermore it seems plausible that a larger fraction of copy number changes is under selection (either positive or negative), whereas the majority of mutations are likely neutral passengers. The balancing estimates on all possible combinations of tumour samples yield results similar to the mutational burden in renal cancer (Fig. 2 panels a2 to j2 and Fig. 3 panels a2 to k2). The majority of subsamples resemble balancing estimates from the full data set. Again, we observe the trend of a bimodal distribution of the balancing factor *f* for small numbers of tumour samples.

We note that our analysis does not depend on the detailed effects of selection, i.e. whether selection acts on copy number changes, mutations or epigenetic alterations. Changes in tree balance caused by any type of fitness advantage could potentially be detected. Moreover, the evolutionary mechanisms that generate balanced or unbalanced trees can be arbitrarily complex^29^. Our method is agnostic to the specific evolutionary dynamics of the tumour, but instead it leverages on the existing data and in particular on the topology of the phylogenetic tree. Our approach is based on the assumption that multi-region profiling represents the tumour’s evolutionary history, e.g. the samples are equally spatially distributed throughout the whole tumour and are not restricted to a small region only.

## Discussion

Accumulating evidence indicates that future personalised treatment strategies of human malignancies must be based on information from multi-region profiling of tumours^8^,^30^. Once multi-region sampling becomes available in routine clinical practice, physicians will have to make informed decisions on how many samples per tumour in the individual patient need to be independently sequenced for optimal therapy. Our study provides a rationale for how many samples are necessary to achieve a certain level of confidence that truly clonal alterations in a tumour have been identified from multi-region profiling. Assigning clonality to specific alterations implies also the identification of sub-clonal alterations. The distribution of sub-clonal alteration contains important information on the evolutionary history of tumours^25^,^27^. However, here we investigated the impact of standard multi-region profiling on treatment decision and focused on clonal alterations. Our method allows tailoring of the number of independent samples that is necessary for each individual tumour. Although the cost of genome sequencing is decreasing rapidly, the prospect of multiple sample profiling in each patient may present a new and daunting financial burden on healthcare systems, especially as the identification of truly clonal alterations in unbalanced tumours (*f* ≪ 0.5) may be difficult and perhaps less cost-effective, posing new challenges. However, in many cases the required number of independently sequenced samples appears surprisingly manageable.

Our approach is independent of any threshold that is often imposed from a statistical analysis of the distribution of mutations identified in a tumour. Our analysis also suggests that the optimal time to perform genome profiling in tumours is at the time of diagnosis since therapy appears to introduce strong selection that may interfere with the identification of the therapeutically relevant truly clonal mutations or immune therapeutic targets^8^,^18^. Tumours at relapse might require denser sampling compared to treatment naive tumours. The list of truly clonal mutations identified by our approach will potentially include tumour driver alterations that could be a targeted for therapy. Although our approach cannot identify a priori the driver mutations, this method will significantly restrict the search for such drivers. This study represents one of many necessary steps to advance from purely descriptive tumour sequencing towards individualized therapies based on quantitative evolutionary principles.

## Methods

### Mathematical model

Let us consider the complete phylogenetic tree of a tumour at a certain time *t* (e.g. at diagnosis). Each leaf of this tree is a cell. Assume there are *N* leaves and therefore *N* − 1 bifurcations in the tree. By definition, alterations present in the trunk of this tree are truly clonal and thus are present in all cells of the tumour. The first bifurcation splits the tumour into two subpopulations, the “left” side with proportion *f*, and the “right” side with proportion 1 − *f*. If we were to take a single tissue sample, many alterations carried by this subpopulation would likely not be truncal. If we took a second tissue sample, we would increase our chance to identify truly clonal alterations. In this case, we have three possibilities: with probability *f*^2^ we have two tissue samples from one side, with probability (1 − *f*)^2^ we have two tissue samples from the other side, and with probability 2*f*(1 − *f*) we have one tissue sample from each side. Only in this last case, the alterations common to both samples would represent the true set of truncal (clonal) alterations and consequently must be present in all cells of the tumour. With *n* independent samples, the probability *p* to have picked both sides of the tumour becomes


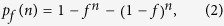


resulting in a non-linear dependence of the probability to find the true set of clonal mutations through *n* samples. A single sample never provides the full information, as *p*_*f*_(1) = 0 for *n *= 1. The expected gain of information with an additional sample *n *+ 1 is


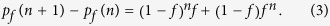


For example consider the case of a perfectly balanced tree (e.g. a neutrally expanding tumour^27^). This implies *f *= 0.5 and the expected gain of information from sample *n* to sample *n *+ 1 is


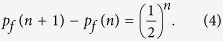


The information gain due to the inclusion of additional samples decreases exponentially, in other words: in the case of balanced trees with *f* ~ 0.5, such as neutral or nearly-neutral trees, relatively few independent tumour samples are needed to identify all true clonal alterations. If we define the remaining uncertainty to have missed the true clonal alterations to be *σ *= 1 − *p*, we can rearrange Equation (2) for the case of a balanced tree with *f *= 0.5 and find the required number of samples *n* necessary for a certain confidence


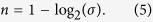


For example, a remaining uncertainty of 1% requires only *n* ≈ 8 independent tumour samples. This level of resolution has already been reached in several recent multi-region sequencing studies^18^,^20^,^23^,^25^ and poses a realistic target for daily clinical care in the near future.

However, one “side” of the tumour could be very small with *f* ≪ 0.5 (i.e. the tumour is highly unbalanced), implying that different parts of the tree have grown at radically different rates, e.g. due to clonal selection. In this case, Equation (2) can be approximated by *p*_*f *→ 0_(*n*) ≈ *nf* and the remaining uncertainty decreases linearly in *n*. For sufficiently small *n*, the gain of information by an additional tumour sample becomes incremental


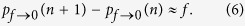


In this case, many tumour samples are required to reach a high level of confidence of finding all true clonal alterations. However, a very slowly growing side contributes very little, if at all, to the overall aggressiveness of the tumour, especially if this side virtually vanishes (*f *→ 0). Although, many samples are needed to infer all true clonal alterations in this situation, the clonal alterations of the extremely dominant and tumour-driving side are of practical interest and again fewer samples may suffice. However, very small ancient sub-clones might drive tumour relapse, as is for example observed in certain leukaemias^31^,^32^.

In general, the remaining uncertainty is given by


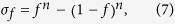


which lies between a linear (*f *→ 0) and an exponential (*f *→ 1/2) gain of confidence with additional samples *n*.

### Data analysis

Here we propose a method to calculate the probability *p*_*f*_(*n*) to find all clonal alterations from *n* independent tumour samples. This method allows us to infer the balancing factor *f* of a tumour with respect to the first bifurcation and thus to estimate the expected gain of information with respect to truly clonal alterations by including additional tumour samples in the analysis:

(i) Collect *n* samples of a tumour.

(ii) Analyse the *n* samples and determine all alterations.

(iii) Take the intersection of all alterations of all *n* tumour samples.

(iv) Take the intersection of all alterations of all possible combinations of 1 to *n *− 1 tumour samples.

(v) Calculate the probability that the alteration identified in step (iii) and (iv) coincide.

By definition, this probability approaches 1 for the combination of all *n* samples.

To allow a comparison with Equation (2), we have to normalise accordingly and get


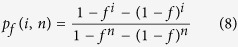


Here, *n* is the maximal number of available samples and *i *= 1,…,*n* denotes possible sub-samples. The only free parameter of this equation is *f*. Thus fitting Equation (8) to actual tumour data allows us to infer *f*, see for example Figs 2 and 3. We use standard least square regression to infer the single free parameter *f*.

Our algorithm is sensitive to misclassified mutations, e.g. mutations not found in a subset of samples due to normal contamination or limitations of sequencing depth (false negatives). Those are inevitable problems in multi-region sequencing studies, leading to a few mutations that seem to contradict the phylogenetic history of these tumours, the so-called “homoplasy” events. Standard phylogenetic reconstruction algorithms, such as Maximum Parsimony, discard those, hence we filtered the few homoplasy events present in a small subset of renal patients (3/10) from our analysis.

## Additional Information

**How to cite this article:** Werner, B. *et al*. Detecting truly clonal alterations from multi-region profiling of tumours. *Sci. Rep.* **7**, 44991; doi: 10.1038/srep44991 (2017).

**Publisher's note:** Springer Nature remains neutral with regard to jurisdictional claims in published maps and institutional affiliations.
